# Duck Productivity in Restored Species-Rich Native and Species-Poor Non-Native Plantings

**DOI:** 10.1371/journal.pone.0068603

**Published:** 2013-07-01

**Authors:** Ryan D. Haffele, Michael W. Eichholz, Cami S. Dixon

**Affiliations:** 1 Cooperative Wildlife Research Laboratory, Southern Illinois University Carbondale, Carbondale, Illinois, United States of America; 2 Cooperative Wildlife Research Laboratory, Center for Ecology, Department of Zoology, Southern Illinois University Carbondale, Carbondale, Illinois, United States of America; 3 Chase Lake National Wildlife Refuge, United States Fish and Wildlife Service, Woodworth, North Dakota, United States of America; University of Western Ontario, Canada

## Abstract

Conservation efforts to increase duck production have led the United States Fish and Wildlife Service to restore grasslands with multi-species (3-5) mixtures of introduced cool season vegetation often termed dense nesting cover (DNC). The effectiveness of DNC to increase duck production has been variable, and maintenance of the cover type is expensive. In an effort to decrease the financial and ecological costs (increased carbon emissions from plowing and reseeding) of maintaining DNC and provide a long-term, resilient cover that will support a diversity of grassland fauna, restoration of multi-species (16-32) plantings of native plants has been explored. We investigated the vegetation characteristics, nesting density and nest survival between the 2 aforementioned cover types in the Prairie Pothole Region of North Dakota, USA from 2010–2011 to see if restored-native plantings provide similar benefits to nesting hens as DNC. We searched 14 fields (7 DNC, 271 ha; and 7 restored native, 230 ha) locating 3384 nests (1215 in restored-native vegetation and 2169 in DNC) in 2010-2011. Nest survival was similar between cover types in 2010, while DNC had greater survival than native plantings in 2011. Densities of nests adjusted for detection probability were not different between cover types in either year. We found no structural difference in vegetation between cover types in 2010; however, a difference was detected during the late sampling period in 2011 with DNC having deeper litter and taller vegetation. Our results indicate restored-native plantings are able to support similar nesting density as DNC; however, nest survival is more stable between years in DNC. It appears the annual variation in security between cover types goes undetected by hens as hens selected cover types at similar levels both years.

## Introduction

Declines in numerous populations of grassland nesting birds are thought to be caused by declines in productivity due to loss of native grasslands [1-7]. The loss of native grasslands has altered the landscape resulting in a fragmented habitat, potentially negatively influencing grassland bird productivity [8]. Additionally, the removal of top predators like gray wolves ( *Canis* *lupis* ) has altered the predator community, allowing meso-predators liked striped skunks ( *Mephitis* *mephitis* ) and red fox ( *Vulpes* *vulpes* ), which efficiently forage for grassland nests, to become the dominant predators, augmenting the negative effect of habitat loss on nest success [9].

The Prairie Pothole Region of North America is an important nesting region for grassland nesting birds, as wetland densities on the breeding grounds produce abundant food sources attracting breeding birds [10]. However, this area has undergone extensive modification due to agricultural development including a loss of >70% of its native grasslands [11,12]. Since the implementation of the North American Waterfowl Management Plan in 1986 numerous conservation organizations including the United States Fish and Wildlife Service (USFWS) have acquired private row cropped fields for the express purpose of providing habitat for nesting ducks. Early studies indicated the mixture of intermediate wheatgrass ( *Thinopyrum* *intermedium* ), tall wheatgrass ( *Thinopyrum* *ponticum* ), alfalfa (*Medicago sativa*), and sweet clovers ( *Melilotus* *spp.*) commonly referred to as dense nesting cover (DNC) is more productive for duck production than the 3-5 species (low-diversity) of native vegetation that were established for comparison when grasslands were initially restored [13,14]. Since the primary purpose of the plantings is to increase duck productivity, these former crop fields have been mostly converted to DNC [15]. While DNC appears to be attractive to ducks, under current conditions of extreme fragmentation and abundant meso-predators, productivity of ducks remains low with nest success often below 20% [16,17]. Additionally, although DNC has been demonstrated to support a number of grassland nesting passerines [18-23], the abundance and diversity of organisms found in DNC is often significantly below that found in native prairie vegetation [7,24-33]. Finally, DNC is typically associated with a cyclic management regime, as it reaches maximum growth in the first 2–4 years after planting, then often loses its structural quality the next 4-5 years as it is outcompeted by species of vegetation that are undesirable to nesting birds. This forces managers to remove the residual vegetation and farm the area for 2-3 years before reseeding DNC [15]. Thus, although DNC appears to achieve the primary purpose of providing quality duck habitat, it does not create a self-supporting ecosystem resilient to perturbation without further assistance; a goal of true restoration [34,35].

More recently wildlife managers have initiated a species-rich approach of native vegetation establishment. This approach differs from the original approach where 3-5 species of vegetation were used in the seed mix by including 16-32 species, a level that reaches a saturation point, thus, is more resistant to invasion of exotic species [36-38]. Similar to DNC, productivity and vegetation vigor within species-rich planted fields declines as the stand ages, however, proper disturbance of native vegetation that is adapted to the local environment and disturbance can eliminate the need to reseed plantings [15,39,40]. Not only will this new approach of species-rich native plantings be more consistent with habitat restoration objectives and USFWS policy, but it should provide habitat for a more diverse group of organisms including more grassland nesting passerines [7,25,26,29,31,32]. This restoration activity is proposed, however, on property specifically acquired to support breeding ducks, thus, the potential influence on duck populations of this new management strategy should be investigated.

The limited benefits to duck productivity of native plantings relative to DNC is most often explained by the limited number of species of vegetation that were previously used in native plantings (3-5 species of warm-season native grasses) [14]. Low diversity plantings are more susceptible to invasion of exotic plant species and provide lower levels of productivity at maturity [38]. More diverse species rich plantings of 16–32 species in the Prairie Pothole

Region resist invasion of exotic species and maintain higher productivity at maturity, thus, should better support nesting birds relative to the fields of low-diversity native vegetation evaluated in the past [38]. A comparison of duck productivity between species rich (16-32 species) native plantings and DNC, however, has not been conducted. Thus, our objective was to compare nest survival and density between the 2 cover types.

## Methods

### Ethics Statement

Field methods were approved by Southern Illinois University Carbondale Animal Care Protocol 08-038 and data was collected under North Dakota Game and Fish permit no. GNF02763149. All study sites were approved by the Devils Lake Wetland Management District, United States of America Fish and Wildlife Service. This field study did not involve endangered or protected species.

### Study Area

**Figure 1:**
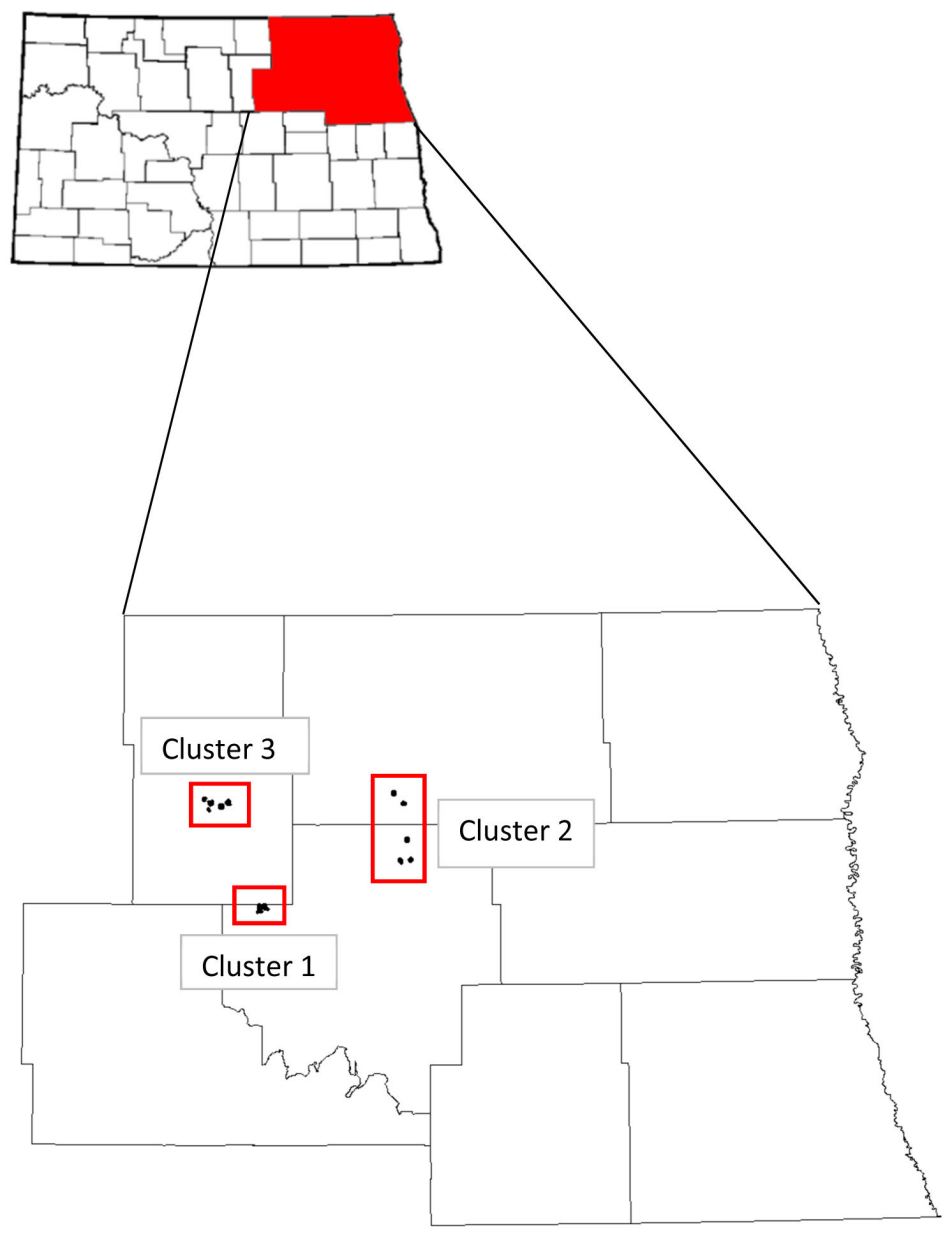
Map of study area. Map of study sites divided into clusters in the Devils Lake Wetland Management District, North Dakota.

The study area was located in the Devils Lake Wetland Management District in northeastern North Dakota. Study fields were located in Ramsey, Towner, and Cavalier counties. We collected data on 14 study fields, 7 planted in DNC and 7 planted with a mixture (17–27 species) of native cool and warm season grasses and forbs; mixtures were based on soil and moisture conditions in each specific field (Table 1). Each field was assigned to a cluster based on geographic location (Figure 1). Up to 2008 (prior to the study), fields were managed by methods commonly used to maintain early successional grasslands (grazing, mowing, and burning), thus maximizing productivity of nesting ducks. As would be expected under normal management practices, type of management action was dependent on what method was deemed most suitable for that particular field. The time period between these management actions and the initiation of our study was adequate to prevent management actions from directly impacting the outcome of our study [22,41-45]. In the fall of 2010, however, a variety of management actions occurred on 6 of 14 study fields. Two of the fields (1 native and 1 DNC) were grazed with cattle from 1 July to 10 August. Two native fields were “clipped” where specific areas within a field with nuisance and exotic species were mowed while the rest of the field was left unmanaged. The other 2 (1 native and 1 DNC) managed fields were hayed. Because we were unable to control for the variation caused by these management actions and because these management actions would have likely directly impacted the outcome of our study, these fields were excluded from all analysis in 2011.

**Table 1:** List of study sites including field name, type of cover, size of field, age of stand (years since field was seeded as of 2010) and the nest success and standard error during 2010-11 field seasons.

**Field Name**	**Cover**	**ha**	**Age of Stand**	**Year**	**Success (%)**	**SE**
Nik Central	DNC	43	6	2010	63.00	6.7
				2011
Register West	Native	40	4	2010	40.46	8.4
				2011
Cami	Native	32	5	2010	59.20	6.3
				2011
Nik South	DNC	13	6	2010	33.04	8.2
				2011
Nik Southeast	DNC	59	18	2010	54.88	5.7
				2011	25.87	4.8
Halvorson	Native	61	16	2010	60.25	8.5
				2011	44.49	10.4
L. A. DNC	DNC	64	6	2010	15.06	2.8
				2011	38.77	5.8
Toilet	Native	22	2	2010	36.80	4.3
				2011
L. A. North	Native	8	6	2010	16.20	4.8
				2011
Mart Native	Native	41	2	2010	59.68	5.2
				2011	14.10	2.0
Mart DNC	DNC	28	7	2010	73.92	4.9
				2011	32.28	4.1
Phil Aus	DNC	38	6	2010	12.78	3.1
				2011	30.68	4.0
Dahl	Native	26	15	2010	71.34	6.4
				2011	3.15	1.6
Weaver	DNC	26	21	2010	67.56	4.9
				2011	50.87	4.5

### Field Sampling

To compare vegetative structure between habitat types, we recorded vegetation data at random locations within each field, with 1 random point being assigned for every 2 ha of the field to ensure sampling throughout the entire field. We overlaid each field with a grid composed of 2 ha blocks and generated random points in each block using Hawths Tools for ArcMap 9.3 (Environmental Systems Research Institute [ESRI], Redlands, California, USA). This resulted in a total of 266 random points; 126 in restored-native plantings and 140 in DNC.

We collected data in two time periods of each study season. The first data were collected in late April before nest searching began to characterize vegetation structure when early nesting species initiated their nests. The second data collection occurred in the middle of June, characterizing vegetation structure for hens who initiated nests late in the nesting season. We determined the vegetation height, visual obstruction (hereafter cover density), and litter depth in the early sampling period and the vegetation height, cover density, and species composition in the late sampling period. We used Robel poles to index the cover density and vegetation height. Cover density readings were taken in the 4 cardinal directions at a distance of 4-m and a height of 1-m by marking a point on the Robel pole where vegetation obscures the pole 100%. These readings gave cover density measurements that are strongly related to the amount of vegetation present, thus giving an indication to the structure available for hens to nest in [46]. We averaged the 4 readings to obtain an overall estimate of the cover density around the nest. We determined the vegetation height to be the point on the Robel pole that > 80% of vegetation was growing below [47]. We measured the litter depth by measuring the height of dead vegetation that forms a mat layer on the ground using a standard ruler in cm [48]. Litter depth was only measured during the early sampling period, as no new litter formed and decomposition was assumed to be negligible between sampling periods. To be classified as litter, vegetation had to be lying on the ground, as we did not measure standing residual vegetation as litter. Vegetation litter provides a suitable nesting substrate for hens and may be important for the concealment and survival of nests [18,49-51]. Species composition was determined using a m^2^, with each species identified being assigned a cover class [52].

To test for differences in nesting density and success, we systematically searched all upland cover in a field for nests starting in the first week of May and concluded searching the first week of July. Each field was searched 7 times on 8 day intervals. Nests were located using teams of 2 dragging a 50-m cable-chain behind all-terrain vehicles (ATV) [53]. Speeds were kept between 3–8 km/h by keeping ATV’s in low gear allowing drivers to stay in a straight line and watch the cable drag [53]. Dragging at speeds faster than 8 km/h increases the likelihood of the chain passing over a nest without flushing the hen. We searched for nests between 0700 and 1400 to maximize the probability of the hen being on the nest [54]. We alternated the starting location of fields for each drag to prevent the same area of the field being searched during the same time of day, reducing the possibility of a hen being on an incubation break during subsequent searches. We marked each nest found with a 1-m wooden lathe painted white with red on the top to allow easy visualization in the field by searchers. The wooden lathe was placed 10-m north of the nest and numbered to give each nest its own unique identification. A 3-mm diameter metal rod painted orange was placed on the north rim of the nest bowl at each nest to assist with relocation. Nests were monitored on 5-8 day intervals until fate was determined (e.g., successful, depredated, abandoned). We determined the clutch size and incubation status at each visit. Incubation status was determined with a simple field candler made from 1-inch radiator hose [55]. We recorded the date, field, species and Universal Transverse Mercator coordinates for each nest. In 2010, we monitored the first 100 nests found in each field, then randomly selected 20 nests from each subsequent search for fields with > 100 nests due to time constraint. In 2011, we monitored all nests found. After each visit, the nests were covered using material from the nest and a marker in the form of an X made out of vegetation was placed on top. If the X was found undisturbed on the next visit, we considered it abandoned due to investigator disturbance and censored it from survival analysis.

### Statistics Analysis

To determine if variation in vegetation type led to differences in cover density, litter depth, and vegetation height between cover types, we analyzed the data using 3 mixed model analysis of variances (ANOVA) in SAS 9.2 (PROC MIXED; SAS 9.2 SAS Institute Inc., Cary, North Carolina, USA, 2008) for each time period separately. For comparison during the early sampling period, cover density, litter depth, and vegetation height were the dependent variables, cover type was the independent variable and cluster was included as a random effect. We included cluster to control for any variation that may have occurred due to geographic differences. A similar analysis with the exclusion of litter depth was conducted for comparison of vegetative structure between habitat types during the late sampling period. We used Shannon’s diversity index to compare alpha and beta diversity between treatments to ensure the treatment of high and low diversity seed mixes was maintained as vegetation matured [56].

To compare daily nest survival and nest success between habitat types we used Dinsmore’s model in Program MARK to estimate daily survival rates (DSR) of nests for each field [57-59]. We assumed a 35-day exposure period to convert DSR to point estimates of nest success and estimated the standard error of point estimates using the Delta Method [53]. To determine if there is evidence for a treatment effect on daily survival rate, we compared a model that included cover type to a model that excluded cover type after accounting for the influence of age of field (number of years since field establishment) using Akaike Information Criteria (AIC) [60]. Because the influence of age of field may differ between the cover types, we included both an additive and interactive model. Furthermore, because previous studies have indicated nest survival increases with field age as vegetation vigor increases but declines as the field ages and vegetation becomes less productive and is invaded by undesirable species, we included a model with a quadratic term for field age. To better explain potential causes of variation in nest survival between habitat types, we also tested for a treatment effect of nest initiation date on DSR in each cover type by comparing the additive (estimating 1 coefficient for both cover types) and interacting (estimating separate coefficients for each cover type) models of initiation date and cover type.

To compare nesting density between habitat types, we estimated the density of nests adjusted for detection probability in each field by taking the total number of nests we found in each field and dividing it by the DSR of the field raised to the power of the average age of the nests found in that field (# of nests found in field/*DSR* ^avg age of nest found in field^) [17]. Correcting for DSR is necessary when estimating density of duck nests because nests are located by disturbing the hen from the nest, thus, only nests that are being actively incubated are located [17]. If DSR varies between habitat types, estimates of nest density will be biased due to variation in detection probability. We used a mixed model ANOVA with the density estimates for each field as the dependent variable, cover type as the independent variable, the amount of wetland shoreline as a covariate and cluster as the random effect. Other studies have found that breeding bird densities are related to wetland densities on the breeding grounds [10]; therefore, we classified wetlands according to Stewart and Kantrud [61] and measured the amount of temporary, seasonal, semi-permanent, and total shoreline in each field. We used these measurements to account for any differences in wetland abundance that may have influenced densities of nesting hens.

To determine if species-specific nesting densities varied between cover types, we used a mixed model ANOVA for each species with species and cover type as the independent variables, density as the dependent variable and cluster as a random effect. We determined species density using the same formula as overall duck nest density.

## Results

We analyzed data from 274 random points located within the 14 study fields in 2010. Vegetation characteristics were not different between cover types during either sampling period (Table 2). In 2011 we analyzed 153 random points from 8 fields. In the early sampling period, there was no difference (F_1,5_= 0.03, p = 0.87) in height between the cover types, as native plantings had an average height of 8.94 ± 0.85 cm and DNC plantings had a height of 10.22 ± 1.61 cm. There was also no difference (F_1,5_= 0.82, p = 0.41) in cover density between cover types with native plantings having an average density of 5.99 ± 0.76 cm and DNC averaging 8.40 ± 1.66 cm. Litter depth was different (F_1,6_= 8.33, p = 0.03) between cover types as native plantings had an average depth of 2.44 ± 0.37 cm and DNC had an average of 5.00 ± 0.63 cm. As expected based on seed mixes, species composition of vegetation varied between cover types, with native plantings and DNC having an average alpha diversity of 22.7 and 13.3, respectively. Native plantings had a beta diversity of 22, while DNC had a beta diversity of 9.

**Table 2:** Average vegetation height (SE), cover density, litter depth, and mixed model analysis of variance results for random locations in multi-species native plantings and dense nesting cover (DNC) during the early and late sampling periods from 2010–2011 in the Devils Lake Wetland Management District, North Dakota.

			**Early Sampling Period**			
	**2010**			**2011**		
**Veg Characteristic**		**DNC**	**Native**		**DNC**	**Native**
Height (cm)		11.94 (1.38)	10.73 (2.20)		10.22 (1.61)	8.94 (0.85)
Cover Density (cm)		6.51 (0.89)	4.22 (0.57)		8.40 (1.66)	5.99 (0.76)
Litter (cm)		4.56 (0.54)	3.24 (0.51)		5.00 (0.63)^a^	2.44 (0.37)^a^
			**Late Sampling Period**			
Height (cm)		28.04 (2.12)	23.82 (2.32)		33.65 (2.97)^a^	19.35 (2.69)^a^
Cover Density (cm)		19.59 (2.92)	15.84 (1.86)		23.63 (4.41)	13.01 (2.68)

^a^ Denotes Significant Difference (p < 0.05)

During the late sampling period, there was a difference in height (F_1,5_= 35.30, p < 0.01) between cover types with native plantings having an average height of 19.35 ± 2.69 cm and DNC having an average height of 33.65 ± 2.97 cm. The difference in cover density was approaching statistical significance (F_1,5_= 4.15, p = 0.10), as native plantings had an average cover density of 13.01 ± 2.68 cm while DNC had an average of 23.63 ± 4.41 cm.

### Nest Density

We located 3,384 nests of 8 species during the 2010-11 field seasons (Table 3). Nest density varied widely between fields ranging from 1.09 nests/ha to 15.06 nests/ha in 2010 and 1.19 nests/ha to 12.05 nests/ha in 2011. Cover type did not have an effect on nest density (F_1,19_ = 0.20, p = 0.66; Table 4), as DNC plantings had an average density of 6.71 (SE = 0.96) nests/ha and native plantings had 6.17 (SE = 1.61) nests/ha for both years combined. The cluster × type interaction was significant (F_2,8_= 4.59, p = 0.05) in 2010, however, no clear pattern was shown as density was higher for native plantings in 1 cluster while the other 2 clusters had higher densities for DNC. The amount of shoreline in each field did not have an effect on nest density (F_1,13_= 1.60, p = 0.25). However, the amount of temporary shoreline was marginally significant (F_1,13_ = 5.00, p = 0.06) in 2010.

**Table 3:** Total number of nests broken down by cover type and species in 2010-11 in the Devils Lake Wetland Management District, North Dakota.

**Cover Type**		**Year**	**Mall^a^**	**BWT^b^**	**Gadw^c^**	**Shoveler^d^**		**Pintail^e^**		**GWT^f^**	**Scaup^g^**	**Wigeon^h^**	**Total**
Native		2010	98	212	254	138		61		7	21	7	798
Native		2011	89	123	102	53		36		4	7	3	417
DNC		2010	126	315	346	234		96		5	65	4	1191
DNC		2011	174	267	309	108		82		0	36	2	978
Total			487	917	1011	533		275		16	129	16	3384

a Mallard

b Blue-winged Teal

c Gadwall

d Northern Shoveler

e Northern Pintail

f Green-winged Teal

g Lesser Scaup

h American Wigeon

**Table 4:** Nest density, adjusted for detection probability (SE), amount of temporary shoreline, and mixed model ANOVA results examining effect of cover type on density in 2010-11 in the Devils Lake Wetland Management District, North Dakota.

**Year**	**CoverType**		**Density (nests/ha)**		***F***	***P***	**Temporary Shoreline (m)**		***F***	***P***
	Native		6.01 (2.11)				2497.61			
2010					0.48	0.51			5.00	0.06
	DNC		5.87 (1.18)				5101.88			
	Native		6.59 (2.75)				1355.75			
2011					0.08	0.79			0.03	0.87
	DNC		7.88 (1.60)				2865.56			

Nest densities were not different between cover types for any species (F_4,99_ = 0.16, p=0.96). Mallard densities averaged 0.99 ± 0.25 nests/ha in DNC and 0.90 ± 0.46 nests/ha in native plantings for both years combined. Pintails had the lowest densities at 0.58 ± 0.10 and 0.45 ± 0.12 nests/ha for DNC and native plantings respectively. Shovelers averaged 1.03 ± 0.10 and 0.78 ± 0.12 nests/ha in DNC and native plantings. Gadwall had an average density of 2.00 ± 0.42 nests/ha in DNC and 1.55 ± 0.58 nests/ha in native plantings. Teal densities in DNC were 1.84 ± 0.37 nests/ha, while native plantings had an average of 1.76 ± 0.46 nests/ha.

### Nest survival

Of the 3,384 nests used in the nest density analysis, we used 2,589 to determine the survival rate for each field. The nests not used in the analysis were censored due to investigator damage (32 nests), abandoned due to investigator disturbance (121 nests), or they were not randomly selected to be monitored in 2010 (222 nests in native fields and 420 nests in DNC nests); all nests were monitored in 2011. The data were best fit by a model that included year and habitat type, with continuous variable for the influence of age of field, including a quadratic term for age of field for each cover type separately. Nest success was variable across fields; 3.15% (1.6) to 73.92% (4.9) (Table 1). To account for model uncertainty associated with the relationship between field age and nest survival (the top 4 models) we used model averaging to estimate nest survival between the cover types and years. In 2010, we estimated model averaged nest success to be 42.3% (7.3) in fields with native vegetation as cover and 44.2% (4.6) in fields with DNC as cover while estimates for 2011 were 10.7% (2.7) for native plantings and 38.5% (4.4) for DNC (Table 5).

**Table 5:** Model selection results based on Akaike’s Information Criterion (AIC), including number of parameters (K) and model weight (w_i_), used to examine the effect of cover type, year, and age of field on nest success in multi-species native plantings and dense nesting cover in 2010-11 in Devils Lake Wetland Management District, North Dakota.

**Model**	**AICc**	**Δ AICc**	**w_i_**	**K**	**Deviance**
Year x Type x Field Age x Field Age^2^ (2)	6367.56	0.00	0.71	8	6351.56
Year x Type x Field Age	6370.94	3.37	0.13	6	6358.93
Year x Type + Field Age	6371.35	3.79	0.11	5	6361.35
Year x Type x Field Age x Field Age^2^ (1)	6372.93	5.36	0.05	7	6358.92
Year x Type	6404.99	37.43	0.00	4	6396.99
Year x Field Age x Field Age^2^	6422.55	54.99	0.00	6	6410.55
Year	6476.73	109.17	0.00	2	6472.73
Field Age	6484.60	117.04	0.00	2	6480.60
Type x Field Age	6486.08	118.52	0.00	4	6478.08
Type + Field Age	6486.50	118.93	0.00	3	6480.50
Type	6520.65	153.09	0.00	2	6516.65
Null	6527.27	159.71	0.00	1	6525.27

(1) Same coefficient for Field Age^2^

(2) Different coefficients for Field Age^2^

**Figure 2:**
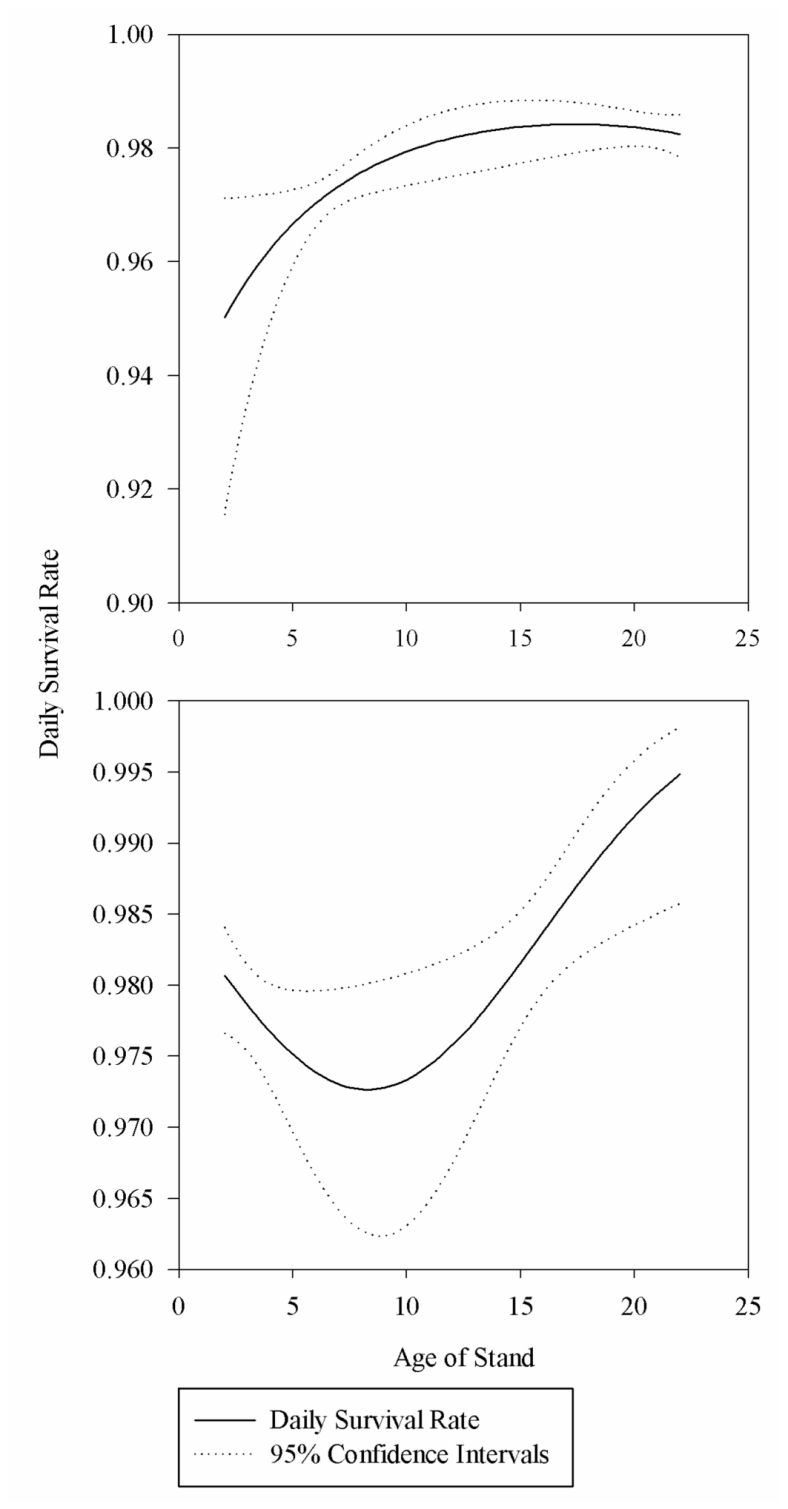
Effect of field age on daily survival rate of nests. Estimated daily survival rate in relation to field age for nests in dense nesting cover (above) and multi-species native plantings (below) from 2010–2011 in the Devils Lake Wetland Management District, North Dakota.

Model comparison indicated both the linear and quadratic form of the relationship between nest survival and field age varied by habitat types. The data were best fit by a model that included separate coefficients for both field age and field age^2^. For DNC, survival gradually increased until approximately 12 years of age then remained at a constant level. In the native vegetation we observed a very different relationship with nest survival decreasing until fields reached approximately 9 years of age at which point they increased (Figure 2).

Because one of our primary interests is the potential for species-rich native vegetation and DNC to support nesting ducks and impacts of field age can be managed by burns or other management practices that “set back” succession, we also estimated nest success for fields without the influence of field age. Without the influence of field age, we estimated nest success to be 48.4% (2.4) in native plantings and 42.4% (2.1) in DNC planting during 2010 and 13.9% (1.7) in native plantings and 37.1% (1.7) in DNC during the 2011 field season.

**Figure 3:**
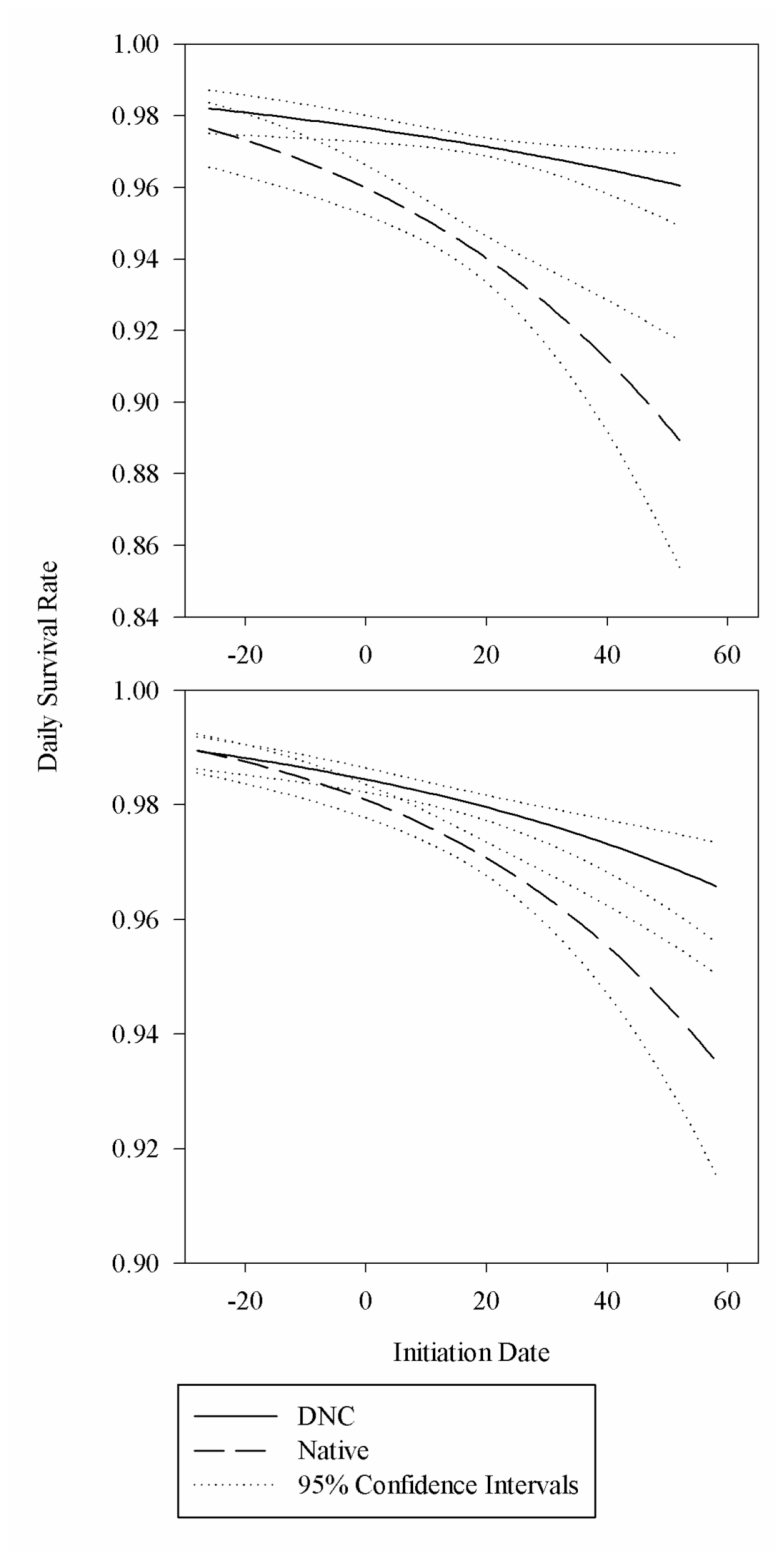
Effect of nest initiation date on daily survival rate of nests. Estimated daily survival rate in relation to nest initiation date for nests in multi-species native plantings and dense nesting cover (DNC) in 2010 (below) and 2011 (above) in the Devils Lake Wetland Management District, North Dakota.

To better understand potential causes of variation in survival between cover types, we also allowed the relationship between nest initiation date and survival to interact with cover type. The data was better fit by a model that included the interaction (Table 6). Nest survival declined as the season progressed with both cover types but the rate was much more dramatic in native vegetation, especially in 2011, the year nest survival differed between cover types (Figure 3).

**Table 6:** Model selection results based on Akaike’s Information Criterion (AIC), including number of parameters (K) and model weight (w_i_), used to examine the effect of nest initiation date and cover type on nest success in multi-species native plantings and dense nesting cover in 2010-11 in the Devils Lake Wetland Management District, North Dakota.

**Model**	**AICc**	**Δ AICc**	**W_i_**	**K**	**Deviance**
Type × Initiation	3794.95	0.00	0.95	4	3786.95
Type + Initiation	3800.65	5.70	0.05	3	3794.65
Cover Type	3821.94	26.99	0.00	2	3817.94
Null	3905.58	110.63	0.00	1	3903.58

## Discussion

### Vegetation Structure Between Cover Types

Our results indicate that species-rich native plantings are able to provide similar vegetation height and cover density as DNC early in the nesting season. Results were variable later in the nesting season as structural characteristics were similar between cover types in 2010 but DNC plantings had taller vegetation in 2011. Litter depths were similar between cover types in 2010 but DNC had more litter in 2011. Our findings contradict previous studies that have shown less diverse native mixes with a component of warm-season species to have taller vegetation than cool-season mixes, but corroborate studies that have found shallower litter depth in warm-season fields [7,29]. The difference in litter depth in 2011 may be a result of the abundance of warm-season species in native fields which are known to remain upright over winter despite snowpack; thus, unlike cool season species which are knocked down by snow cover, would not be included in our estimate of litter [26,62]. Snowpack was likely heavier and denser in 2011, causing the exotic cool season grasses in DNC to be lodged more easily, creating deeper litter depths than in native fields.

Our results also indicated vegetation was taller in DNC during the late 2011 sampling period. We hypothesize the difference in height in the late sampling period of 2011 is due to a disparate influence of cold spring temperatures on warm-season grasses. In 2010, an early spring occurred with April and May temperatures being the warmest since 1981. Conversely, in 2011 temperatures were 10 degrees cooler with above normal precipitation [63], which may have limited growth of warm-season plants. Warm-season species (C_4_) typically begin active growth in early summer when temperatures warm compared to cool-season species (C_3_) that actively grow in the wetter, cooler spring [64]. With the late warming, the warm-season species may have not started active growth until later in the season resulting in a difference of height from the cool-season dominated DNC plantings at the time of the late sampling. While native plantings did include a cool-season component providing earlier growth in these fields, they did not appear to provide the same cover as the cool-season species in DNC.

It is possible the observed difference in vegetation structure between native and DNC is not due to inherent differences between vegetation types but due to the stage of vegetation between the vegetation types in this study. Fifty-seven percent of native fields were planted within 5 years of the initiation of this study while the establishment of native vegetation often isn’t complete for 3-6 years after it is first planted [65-67] (Table 1). The establishment stage for native plantings is usually associated with increased weeds and may not reflect the long term vegetation characteristics of the stand [66,67]. In contrast, all DNC plots had been established for at least 6 years and most (71%) were 6-7 years old, the stage vegetation in DNC is most productive. It is possible fields with native cover were more susceptible to annual climatic variation or variation in predator abundance than DNC because this study took place during the establishment phase.

### Nest Densities

Previous studies comparing duck nesting density between historic low-diversity native cover and DNC have had mixed results. Rodriguez [14] and Kaiser et al. [13] found higher densities in DNC than native warm and cool season grasses. Alternatively, other studies have found no difference between DNC and native plantings [17,68]. Variation in results was likely due to spatial and temporal variation and variation in the proportion of legumes in the plantings. Because all niches within a field are not utilized by the 3-5 species of native vegetation in species-poor native plantings, species-poor native plantings are highly susceptible to invasion by exotic vegetation commonly avoided by nesting ducks [38]. This susceptibility to invasion varies by geographic area and time since planting [38]. Additionally, the proportion of legumes in the seed mix of both native plantings and DNC influences density of nesting ducks [17].

Our study is the first to compare duck nesting density between DNC and species-rich native plantings, native plantings of both warm and cool season vegetation with adequate diversity to saturate the available niches and decrease or prevent the variability among sites caused by the invasion of exotic species [69,70,71,72]. Our results indicate no statistically significant difference in the density of nesting ducks between species-rich native plantings and DNC. Additionally, providing a diverse habitat containing multiple species of cool and warm-season grasses as well as forbs provides greater benefit to a more diverse community of avian species than monocultural stands [24]. Cool-season native grasses start actively growing in the early spring, providing cover and concealment early in the nesting season. Once temperatures warm later in the spring and early summer, these grasses become dormant and warm-season grasses start actively growing [64], providing additional cover and concealment. The addition of native forbs and legumes not only reduces niche availability for unwanted exotic species but provides structural diversity within the stand as they tend to branch out laterally, helping to restrict mammalian predator movement [73].

An additional concern with DNC is the belief that its benefits don’t reach the full spectrum of upland nesting ducks but only benefit species that prefer tall, thick, dense cover like mallards and gadwalls [22,23]. Native mixtures have been found to benefit a greater array of species, especially teal, pintails, and shovelers [13,42]. Our results indicated the majority of species tended to nest in slightly higher densities in DNC; however, no differences were statistically significant. Gadwalls nested in the highest density of any species in DNC, while teal nested in the highest density in native plantings. In contrast to previous findings, DNC in our study supports equivalent densities for species that nest in both dense and sparse cover and native plantings are able to support comparable densities as DNC for all species.

Despite a lack of significant differences in vegetation characteristics between cover types during most sampling periods, trends in vegetation characteristics and species-specific nest densities suggest the non-significant differences may be biologically important. Vegetation tended to be taller and denser with deeper litter in DNC. Additionally, species-specific nest densities tended to be greater in DNC, suggesting the slight difference in vegetation characteristics may be detectible by nesting hens. Depending on the management objectives, however, it is likely other benefits of native plantings, like increased grassland faunal diversity, may outweigh the non-significant differences in species-specific nesting densities between cover types.

### Nest Survival between Cover Types

In contrast to previous findings of similar nest survival when comparing species-poor plantings and DNC, cover type (i.e., species-rich native vs. introduced species) influenced daily survival rates of nests during this study with native plantings and DNC producing similar survival levels in 2010 but DNC having substantially higher nest survival than native plantings in 2011. As previously discussed, past studies were conducted on fields using species-poor native plantings; in contrast, our study fields were composed of species-rich native plantings. Although nest survival declined in both cover types during 2011, the sharp decrease in nest survival for fields with native cover relative to DNC suggests current seed mixtures are more susceptible to temporal variability than DNC. The factor limiting survival in 2011 may be due to variation in vegetation characteristics between field types or a result of a difference in annual variation in predator abundance and distribution between cover types.

In 2011, native plantings did not provide the same vegetation characteristics as DNC. In the early sampling period, litter depth was shallower in native vegetation relative to DNC (Table 2). The difference in litter depth was not likely important, however, as nest survival was similar between cover types early in the nesting season in 2011, when litter depth is most likely to influence survival of nests (Figure 3). The standing residual warm-season grasses in native plantings likely provided the same benefit as leaf litter in DNC, providing cover and concealment to early nesting species. Additionally, in the 2011 late season sampling period, vegetation in the species-rich native plantings was significantly shorter than DNC vegetation. We believe it is unlikely, however, that the difference in vegetation height alone explains the difference in nest survival between cover types in the 2011 breeding season. First, vegetation height was not found to be influential on nest survival after accounting for vegetation density [74]. Furthermore, if annual climatic variation in vegetation growth had caused differences in nest success, we would predict native vegetation in the early and late sampling periods of 2010 would be higher than native vegetation in the early and late sampling periods of 2011 and lower nest survival earlier in the season in years when growth of warm season grasses was delayed by cool temperatures. In contrast to these predictions, we found cover height and density in native habitat were similar between 2010 and 2011, and nest survival was similar between cover types early in the nesting season and decreased thereafter, with native plantings having lower survival later in the nesting season than DNC. Other investigators have found a similar relationship between stage of nesting season and nest survival and proposed the decrease is due to predators responding to changing small mammal and insect populations or due to predators changing foraging patterns as the season progresses [17,27,75,76]. This explanation is consistent with an observation of variation in predator abundance between 2010 and 2011. In 2011 there was a substantial increase in the abundance of nest predators in eastern North Dakota. The population of primary nest predators in the region (skunk, fox, and raccoon [ *Procyon* *lotor* ]) increased 67%, 53%, and 79%, respectively, from 2010 to 2011 [S. Tucker, North Dakota Game and Fish Department, unpublished data]. Combined, these results suggest changes in nest survival between the 2 years of the study were more likely due to changes in predator abundance or distribution than changes in vegetation structure caused by climatic variability; although it is plausible the 2 factors interacted. We found vegetation height to have little impact on nest survival after accounting for the influence of vegetation density; however, the significant difference in vegetation height between species-rich native vegetation and DNC observed late in the nesting season of 2011 and the non-statistically significant difference in native vegetation density and height between 2010 and 2011, may have had a biological influence when predator abundance increased, disproportionately decreasing survival of nesting hens in the native cover late in the nesting season. The higher concentration of predators in 2011 may have more easily exposed the differences in nest survival caused by differences in vegetation structure, resulting in a significant difference in nest survival between cover types.

An alternative and we believe more likely explanation for the difference in nest survival between the 2 cover types in 2011 is the ability of the more diverse native vegetation to support a larger, more diverse animal community, increasing predator abundance in native fields. Native plantings provide more heterogeneous habitat than DNC. This heterogeneity likely created more suitable habitats for alternative prey, especially small mammals [73,77,78]. If native plantings supported more alternative prey, nest predators may have responded resulting in a more dramatic increase in predation of nests relative to DNC as generalist predators became more abundant [79-83]. Alternatively, characteristics of native vegetation might be preferred relative to DNC for nest predators, thus, a response of alternative prey may not be needed as an intermediary and native vegetation might directly attract a greater density of nest predators regardless of abundance of alternative prey.

The native plantings in this study were still in the establishment phase of development, and based on our results did not provide the same structural characteristics or nest security as DNC. Understanding and possibly alleviating the temporal variation in nest survival for native plantings is critical as land managers continue to make decisions on using DNC or native seed mixtures. Nest survival between the 2 cover types was similar early in the nesting season but declined much more dramatically in the species-rich native vegetation late in the nesting season. Research identifying the cause for this temporal variation in the interaction between habitat type, stage of nesting season, and nest survival is critical information that managers need to make decisions that provide optimal habitat for waterfowl nest survival.
